# Signal 3 Cytokines as Modulators of Primary Immune Responses during Infections: The Interplay of Type I IFN and IL-12 in CD8 T Cell Responses

**DOI:** 10.1371/journal.pone.0040865

**Published:** 2012-07-17

**Authors:** Selina Jessica Keppler, Kerstin Rosenits, Tamara Koegl, Smiljka Vucikuja, Peter Aichele

**Affiliations:** 1 Department of Immunology, Institute for Medical Microbiology and Hygiene, University of Freiburg, Freiburg, Germany; 2 Faculty of Biology, University of Freiburg, Freiburg, Germany; University of Georgia, United States of America

## Abstract

Signal 3 cytokines, such as IL-12 or type I IFN, support expansion and differentiation of CD8 T cells *in vivo*. If and how these two signal 3 cytokines compensate each other in T cell activation during different infections is so far unknown. Using CD8 T cells lacking receptors for IL-12, type I IFN or both, we show that the expansion of CD8 T cells depends on type I IFN (LCMV infection), type I IFN and IL-12 (*Listeria* and vesicular stomatitis virus infection) or is largely independent of the two cytokines (vaccinia virus infection). Furthermore, we show that CD8 T cells lacking IL-12 and type I IFN signals are impaired in cytokine production and cytolytic activity in the context of VSV and *Listeria* infection. These effector CD8 T cells fail to express KLRG1, thereby exhibiting a memory-like phenotype which correlated with lower expression of the transcription factor T-bet and higher expression of Eomes. This indicates that the variable interplay of both signal 3 cytokines is mandatory for cell fate decision of CD8 T cells in the context of different infections. Furthermore our results demonstrate that the pathogen-induced overall inflammatory milieu and not the antigen load and/or the quality of antigen presentation critically determine the signal 3 dependence of CD8 T cells.

## Introduction

Activation of CD8 T cells depends on three signals: TCR engagement (signal 1), costimulation (signal 2) and an inflammatory stimulus (signal 3) via cytokines such as interleukin 12 (IL-12) or type I interferons (type I IFN). Both signal 3 cytokines have been shown to support expansion and effector functions of CD8 T cells *in vitro* [1], [2], [3]. In addition, upon *in vitro* stimulation of CD8 T cells in the presence of either IL-12 or type I IFN, CD8 T cells exhibited a comparable gene expression profile. Furthermore, both cytokines facilitated continued gene expression relevant for CD8 T cell differentiation by chromatin remodeling via histone acetylation [4]. *In vivo*, expansion and survival of CD8 T cells is strictly dependent on a direct type I IFN signal during lymphocytic choriomeningitis virus (LCMV) infection but less critical after vaccinia virus (VV) or *Listeria monocytogenes* infections [5], [6], [7]. It was speculated that IL-12, produced during VV and *Listeria* infections, replaces type I IFN as third signal, however a mechanistic proof is still lacking [5], [8]. During LCMV infection the compensatory effect of IL-12 was not observed, since high levels of type I IFN suppress the production of IL-12 [9], [10]. On the other hand, direct IL-12 signaling was mandatory for T cell expansion after *Listeria* infection but not after viral infections with LCMV, VSV or VV [11]. It is not clear whether type I IFN substitutes for the function of IL-12 in CD8 T cell expansion during viral infections. Thus, the redundant role of these signal 3 cytokines for T cell activation during infections is not yet fully understood.

During the early phase of infection CD8 T cells differentiate into short-lived effector cells (SLEC) and memory-precursor effector cells (MPEC), also called effector and memory cytolytic T-lymphocytes (CTL). These effector T cell subpopulations can be distinguished according to their surface marker expression. SLEC (or effector CTL) express high levels of KLRG1 and low levels of CD127 [12] and are not able to establish memory after clearance of the infection. In contrast, MPEC (or memory CTL) that express low levels of KLRG1 and high levels of CD127 survive and form memory cells [12], [13]. The signals required for differentiation of SLEC and MPEC are a matter of debate and several cytokines (IL-12, type I IFN and IL-2) seem to be involved [8], [11], [12], [14], [15], [16]. On the transcriptional level, the differential regulation of the T-box transcription factors T-bet and eomesodermin (Eomes), amongst others, was shown to be essential for this cell fate decision [12], [17], [18]. Both T-bet and Eomes control IFN-γ expression and the generation of cytolytic functions in CD8 T cells. Thereby, T-bet has been suggested to induce the transition of CD8 T cells into SLEC, whereas Eomes expression was associated with memory formation of T cells [18], [19]. It has been proposed that IL-12 induces T-bet and at the same time represses Eomes during *Listeria* infection [19]. However, the causal link between signal 3 signaling and the differential expression of these transcription factors leading to the transition of SLEC versus MPEC is unknown. Furthermore, it is not clear if and how IL-12 and type I IFN substitute each other as signal 3 in different infections. To investigate a possible redundant role of IL-12 and type I IFN as signal 3, we examined T cell responses in the context of four infections (LCMV, VV, VSV and *Listeria)* using CD8 T cells with one defined antigen-specificity lacking receptors for IL-12, type I IFN, or both in an adoptive transfer system. Results reveal a complex pattern of signal 3 dependence of T cells for activation, expansion and cell fate decision in the different infections, with CD8 T cells being either largely independent or differentially dependent on one or both signal 3 cytokines. Although type I IFN can substitute IL-12 signals for expansion and effector functions in some infections, IL-12 predominantly determines high expression levels of the transcription factor T-bet, and therefore regulates the cell fate decision between SLEC and MPEC. Furthermore by using experimental approaches to separate antigen-specific signal 1 from the cytokine milieu, our results demonstrate that the pathogen-induced overall inflammatory milieu and not the antigen load and/or the quality of antigen presentation critically determines the signal 3 dependence of CD8 T cells.

## Materials and Methods

### Ethics Statement

Animal care and use was approved by the Regierungspraesidium Freiburg (G-08/93).

### Mice

C57BL/6 (B6) mice were obtained from Harlan Winkelmann, IL-12Rβ2-deficient (IL-12RKO) (Il12rb2^tm1Jm^) mice and IFN-α receptor deficient IFNARKO (B6.129S7-*Ifnar*1^tm1Agt^) from Jackson Laboratory or Dr. U. Kalinke (Hannover) [20], [21]. P14.WT TCR transgenic (B6.D2-Tg(TcrLCMV)327Sdz/JDvsJ), P14.IFNARKO, P14.IL-12RKO and P14.DOKO mice on a B6.Thy1.1 background were generated by breeding. H8 mice expressing the GP33 epitope of LCMV ubiquitously as a transgene were described [22]. Mice were kept under specific pathogen-free conditions and used at 8–16 weeks of age.

### Infections

LCMV WE and LCMV8.7, recombinant vaccinia virus (rVV_GP_) and recombinant vesicular stomatitis virus (rVSV_GP_) [23] expressing the LCMV glycoprotein were grown on L929 (ATCC number: CCL-1) or BSC40 (derivate of BS-C-1, ATCC number: CRL-2761) cells, respectively. Recombinant *Listeria monocytogenes* (r*Listeria*_GP33_) [24], expressing the LCMV glycoprotein epitope gp33-41, were grown in TSB medium. Mice were infected with 200 PFU of LCMV-WE, 2×10^6^ PFU of rVV_GP_ or rVSV_GP_, and 2×10^4^ CFU of r*Listeria*_GP33_ i.v.

### Adoptive Transfer Experiments

P14 T cells express a transgenic TCR specific for the LCMV glycoprotein epitope gp33-41 in the context of H-2 D^b^. P14 T cells that lack either the type I IFN receptor (P14.IFNARKO), the IL-12 receptor (P14.IL-12RKO) or both (P14.DOKO) were generated by crossing. Spleen cells from donor mice were purified using a negative CD8 purification kit (Miltenyi Biotec), according to the manufactureŕs instructions. Equal numbers (1×10^5^ in all experiments, except Fig. S1 (1×10^4^)) of donor T cells were transferred i.v. into sex-matched B6 mice. After adoptive transfer of P14 T cells, recipient mice were infected with LCMV, rVV_GP_, rVSV_GP_ or recombinant r*Listeria*_GP33_, all expressing the epitope gp33-41. P14 T cells were traced in recipients using anti-Thy1.1 monoclonal antibody.

For BrdU incorporation experiments, recipient mice were treated with 2 mg BrdU i.p. at day 4 after infection.

To expand CD8 T cells in H8 mice independently of an infection, P14.WT and P14.IFNARKO T cells were adoptively transferred into H8 mice. One day after transfer, recipient H8 mice were treated with 6 µg anti-CD40 antibody (clone FGK45, [25]) by intraperitoneally injection as described previously [26] and infected with LCMV8.7 as indicated. Mice were again treated with 6 µg anti-CD40 at day 2 after infection.

### Flow Cytometry

All antibodies were purchased from eBioscience. For analysis of intracellular cytokines, 10^6^ lymphocytes per well were stimulated with 10^−7^ M LCMV gp33-41 peptide in the presence of Brefeldin A for 4 h, followed by surface staining for CD8 and Thy1.1 and intracellular staining for IFN-γ and TNF-α using the Cytofix/Cytoperm kit (BD Bioscience). Intracellular staining for T-bet and Eomes was done directly ex vivo. Staining for incorporated BrdU was done using the BrdU staining kit (BD) according to the manufactureŕs instructions.

All flow cytometry was analyzed on a FACSCalibur or FACSCanto (BD).

### Detection of Cytokines

For detection of the IL-12 and type I IFN levels, mice were infected with either LCMV WE, rVV_GP_, rVSV_GP_ or recombinant r*Listeria*_GP33_ as described. Infected mice were sacrificed 12, 24, 48, 72 and 96 hours after infection. Blood sera were obtained by centrifugation.

IL-12 levels in sera and spleen homogenates were analysed using ELISA (Mouse IL-12p70 Quantikine ELISA kit, R&D Systems). Type I IFN levels were detected by VSV protection assay based on a protocol by Vogel et al. [27].

### Chromium Release Assay

Cytolytic activity of transferred P14 T cells was determined in a standard ^51^Cr release assay using gp33-41 peptide loaded EL-4 cells as target cells or adeno peptide loaded EL-4 cells as a control. Five days after infection with rVSV_GP_ or r*Listeria*_GP33_, P14.WT, P14.IFNARKO, P14.IL-12RKO and P14.DOKO effectors were isolated from whole spleen cell suspensions using Thy1.1-PE antibody staining and anti-PE magnetic bead separation as described above. Effector and target cells were incubated together for 5 hours at 37°C. Duplicate wells were assayed for each E:T ratio starting with a ratio of 10∶1 (10^5^ effector cells to 10^4^ target cells) and % specific lysis was calculated.

### Statistical Analysis

Statistical analysis was determined by one-way ANOVA followed by Bonferroni’s multiple comparison post-test or Student’s unpaired t-test. P values p<0.01 was considered as significant.

## Results

### The Impact of IL-12 and Type I IFN as Signal 3 on CD8 T Cell Responses Varies in different Infections

To examine the impact and possible compensatory effects of IL-12 and type I IFN as signal 3 to CD8 T cell responses in the context of infections, P14 T cells that lack either the type I IFN receptor (P14.IFNARKO), the IL-12 receptor (P14.IL-12RKO) or both (P14.DOKO) were adoptively transferred in B6 recipient mice. Thereafter recipient mice were infected with LCMV, recombinant vaccinia virus (rVV_GP_), recombinant vesicular stomatitis virus (rVSV_GP_) or recombinant *Listeria monocytogenes* (r*Listeria*_GP33_) all expressing the GP33 epitope.

**Figure 1:**
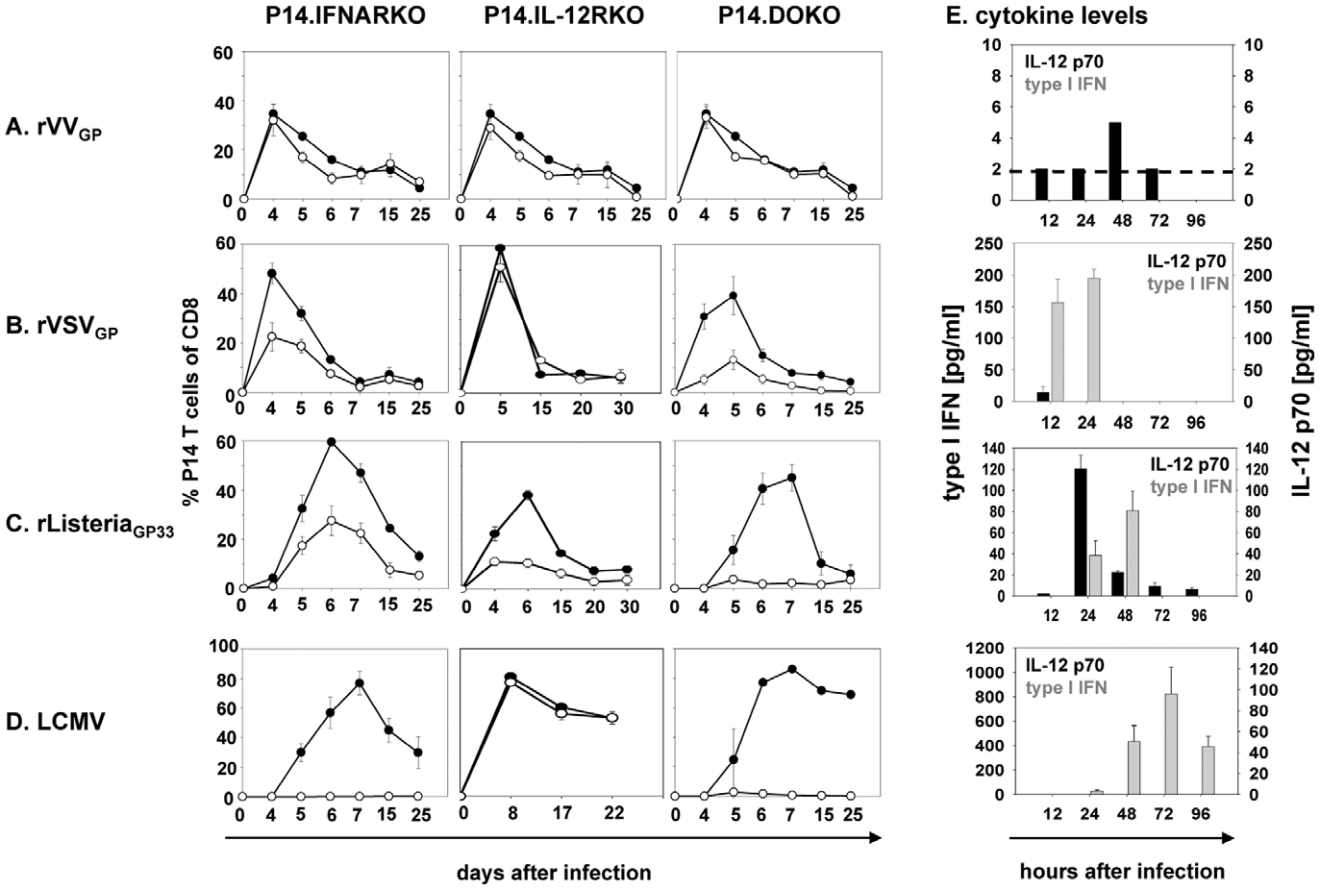
Expansion of CD8 T cells shows a complex pattern of dependency on signal 3 cytokines. 10^5^ (Fig. 1 ***A–D***) Thy1.1^+^ P14.WT, P14.IL-12RKO, P14.IFNARKO or P14.DOKO T cells were transferred into B6 mice (Thy1.2^+^) followed by infection with rVSV_GP_, rVV_GP_, LCMV or r*Listeria*_GP33_. Kinetics of P14.WT (•) and P14.IFNARKO, P14.IL12RKO as well as P14. DOKO (○) T cells gated on CD8^+^ T cells in the blood at indicated time points. Values are expressed as mean ± SEM (n = 3). Results are representative of three independent experiments. IL-12 and type I IFN levels in sera of infected mice as determined by ELISA (for IL-12) or VSV protection assay (for type I IFN) are indicated (Fig. 1 E).

After adoptive T cell transfer and infection of the recipient mice with rVV_GP_, P14.WT, P14.IFNARKO and P14.IL-12RKO T cells initially expanded equally well (Fig. 1 A). After day 4, the T cell populations lacking one of the signal 3 receptors declined slightly more pronounced when compared to P14.WT T cells but formed memory populations of comparable size. Most surprisingly, expansion of P14.DOKO T cells, deficient in both signal 3 pathways, was not limited after rVV_GP_ infection and memory T cells formed independently of a signal 3. Analysis of IL-12 and type I IFN expression in the early phase of VV infection revealed rather low levels of IL-12 and hardly any type I IFN (Fig. 1E). Therefore, after VV infection, neither IL-12 nor type I IFN is required for the expansion of CD8 T cells indicating that T cells are either largely independent of a signal 3 or alternatively, a further signal 3 cytokine has to be postulated.

In the context of rVSV_GP_ infection, in which IL-12 was only detectable in borderline levels at a very early time point and type I IFNs levels were considerably increased (Fig. 1E), P14.WT and P14.IL-12RKO T cells underwent vigorous expansion, reaching a peak at day 4–5, whereas P14.IFNARKO T cells showed diminished expansion, with PBL frequencies reduced by a factor of 2 when compared to P14.WT T cells (Fig. 1 B). These results indicate that type I IFN replaces IL-12 as a signal 3 for T cell expansion, but IL-12 is only partially able to substitute type I IFN signals during VSV infection. Importantly, if both signal 3 cytokines are missing, T cells (P14.DOKO) were drastically impaired in their expansion, with T cell frequencies in PBL reduced by a factor of ∼4 compared to P14.WT T cells (10% versus 40% P14 T cells of total CD8, Fig. 1 B).

In response to infection with r*Listeria*_GP33_, IL-12 and type I IFN were produced (Fig. 1E) [7], [28]. After adoptive transfer and infection with r*Listeria*_GP33,_ both P14 T cells lacking either type I IFN or IL-12 receptor exhibited reduced expansion at day 6, with frequencies in PBLs reduced by a factor of 2 (P14.IFNARKO) to 3 (P14.IL-12RKO) compared to P14.WT T cells (Fig. 1 C). CD8 T cells that lack both receptors (P14.DOKO) showed further impaired expansion (Fig. 1 C) with frequencies reduced by a factor of ∼20 compared to P14.WT T cells (2% versus 40% P14 T cells of total CD8) indicating that both signal 3 cytokines act on *Listeria*-specific T cells. Of note, the peak of expansion of P14.DOKO T cells was already at day 5 (Fig. 1 C).

As previously mentioned and shown in Fig. 1E, LCMV infection induces a strong type I IFN response that suppresses IL-12 production [9], [10]. Hence it was not surprising that CD8 T cells were highly dependent on type I IFN signals for expansion. T cells lacking type I IFN signaling (P14.IFNARKO and P14.DOKO) therefore exhibited drastically reduced expansion after LCMV infection (Fig. 1 D) with frequencies of P14.IFNARKO and P14.DOKO T cells reduced by a factor of 30–40. In contrast, P14.WT and P14.IL-12RKO T cells showed vigorous expansion in the blood, reaching a peak by day 8 (Fig. 1 D). Thereafter both cell populations declined and generated memory populations of comparable size. Despite the severely impaired expansion of P14.IFNARKO and P14.DOKO T cells, some T cells survived and were detectable up to 25 days after infection.

We transferred fairly high cell numbers of P14 T cells (10^5^) in order to obtain enough cells for analysis. Since high input numbers of TCR transgenic T cells may influence T cell differentiation and potentially the dependence of T cells on signal 3, we performed transfers with more physiological P14 T cell numbers (10^4^). Under low precursor frequency conditions, P14.WT and P14.DOKO T cells exhibited the same signal 3 dependence in the context of the infections used compared to our findings after high cell number transfer (Fig. S1). This is in line with our recent publication where consistent results were obtained with low and high numbers of IL-12 receptor deficient P14 T cells and polyclonal T cells in the context of *Listeria* infection [11].

In summary, our results demonstrate a variable requirement for signal 3 cytokines in T cell expansion in the context of different infections that correlated very well with the availability of these two cytokines in the early phase of T cell activation. We next analyzed the influence of type I IFN and IL-12 on CD8 T cell differentiation in more detail. As neither IL-12 nor type I IFN strongly affected the expansion of CD8 T cells during VV infection, and after LCMV infection cell numbers are too low to analyze, we focused on CD8 T cell responses during VSV and *Listeria* infection.

### Interplay of IL-12 and Type I IFN during VSV and Listeria Infection: Impact on Activation and Effector Function of CD8 T Cells

**Figure 2:**
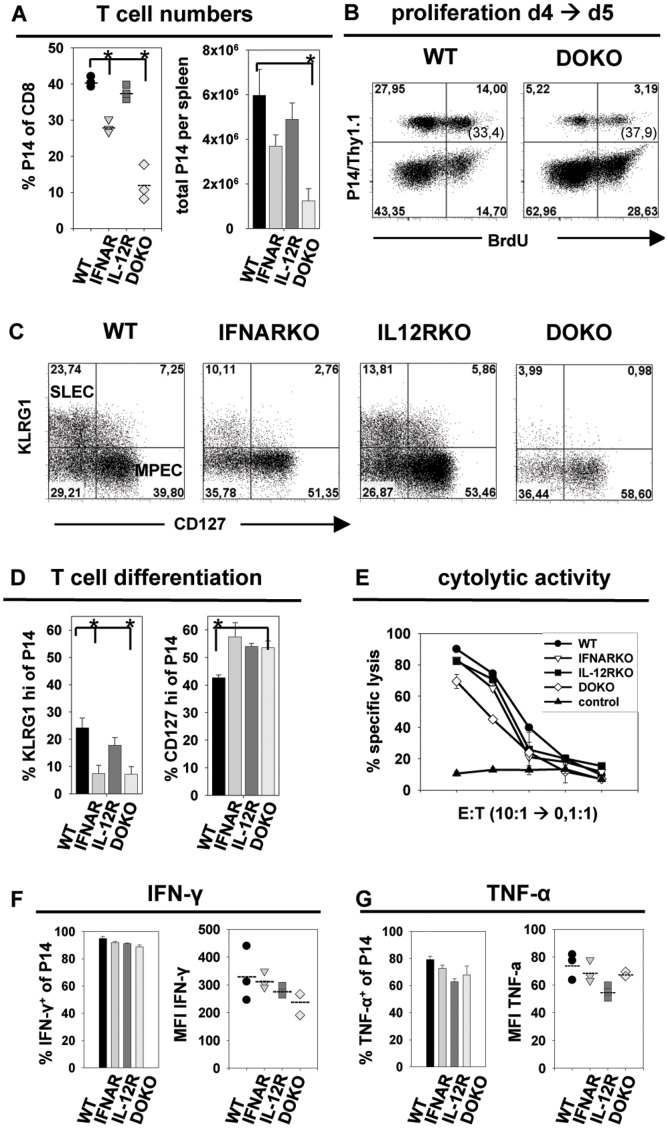
Analysis of P14 CD8 T cells in the context of a rVSV_GP_ infection. P14.WT, P14.IL-12RKO, P14.IFNARKO or P14.DOKO T cells were transferred into B6 mice followed by infection with rVSV_GP_. Analysis of spleen cells at day 5 after infection is shown. ***A***, Relative and total P14 T cell numbers per spleen is shown. ***B***, Analysis of cell division by BrdU incorporation. After adoptive transfer, recipient mice were treated with 2 mg of BrdU i.p. on day 4. BrdU staining of P14.WT or P14.DOKO T cells gated on CD8^+^ T cells on day 5 in the spleen is shown. Numbers in brackets indicate percentages of BrdU^+^ cells of total P14 T cells. ***C***, Analysis of KLRG1 and CD127 expression on P14 T cells. Plots are gated on CD8^+^, Thy1.1^+^ (P14) cells. ***D***, Statistical analysis of T cell differentiation. Percentages of KLRG1 and CD127 positive P14 T cells of total P14 T cells are depicted. ***E***, Cytolytic activity of P14 T cells was determined five days post infection by a ^51^Cr-release assay. Gp33-41 peptide-loaded or adeno peptide-loaded EL-4 cells (control) were used as targets. Equal numbers of purified P14 T cells were used as effectors. Expression of IFN-γ ***(F)*** and TNF-α ***(G)*** of P14 T cells. Percentages of cytokine positive P14 T cells of total P14 T cells, as well as MFI of cytokine expression are indicated. Values are expressed as mean ± SEM (n = 3). *p<0,01. Results are representative of three independent experiments.

After VSV infection, CD8 T cells deficient in IL-12 signaling expanded comparable to wild-type T cells, whereas T cells that lack type I IFN or both cytokine signals were limited in their expansion (Fig. 1 B). For a detailed analysis, the four T cell populations were transferred into B6 mice followed by infection with rVSV_GP_. T cells were examined at day 5 after infection. P14.WT, P14.IL-12RKO T cells showed extensive expansion in the spleen as observed in relative and total cell numbers (Fig. 2 A). In contrast, P14.IFNARKO and even more pronounced P14.DOKO T cells were impaired in their expansion, constituting only about 30% or 11% of total CD8 T cells (versus 40% of P14.WT T cells) in the spleen respectively (Fig. 2 A). The reduced expansion could be due to impaired proliferative capacity of the P14.DOKO T cells at this time point of infection. However, BrdU incorporation experiments revealed that about 35% of all P14.WT and P14.DOKO T cells underwent cell divisions between day 4 and day 5 after VSV infection (Fig. 2 B), indicating a comparable proliferation rate of the two T cell populations. This suggests that impaired survival and not reduced proliferation might be the reason for lower P14.DOKO T cell numbers. As discussed in our previously published paper [11] apoptosis of T cells is difficult to detect by *ex vivo* analysis, since dying cell are immediately removed. Annexin V stainings, caspase 3 assays and analysis of pro- and anti-apoptotic molecules in qRT-PCR did not reveal significant difference in apoptosis between T cell sufficient or deficient in signal 3 *ex vivo*. At the moment, we can not exclude the possibility that the two P14 T cell populations differ slightly in their proliferative capacity at very early time points of clonal expansion. Further analysis of the expression of surface markers KLRG1 and CD127 showed that only about 7% of the P14.IFNARKO and P14.DOKO T cells up-regulated KLRG1. In comparison, 20–25% of P14.WT and P14.IL-12RKO T cells showed high KLRG1 expression. KLRG1 expression therefore correlated with the expansion capacity of the different CD8 T cells. Interestingly, only P14.WT T cells strongly down-regulated CD127, whereas P14.IFNARKO, P14.IL-12RKO and P14.DOKO T cells showed high expression levels of CD127 (about 55% CD127^hi^ cells compared to 40% CD127^hi^ of P14.WT) (Fig. 2 C and D). In addition, all four P14 T cell populations expressed the activation marker CD44 and demonstrated comparable down-regulation of CD62L (Suppl. Fig. 2 A) in the context of a rVSV_GP_ infection. Hence, CD8 T cells that lack a signal 3 during VSV infection exhibited a more MPEC phenotype (KLRG1^lo^/CD127^hi^).

To determine whether impaired expansion of P14.DOKO T cells and the preferential differentiation into MPEC after VSV infection was accompanied by differences in effector functions, expression of IFN-γ, TNF-α and IL-2 as well as cytolytic activity were analyzed (Fig. 2 E, F and G, Fig. S2 A). About 90% of P14.WT, P14.IFNARKO, P14.IL-12RKO and P14.DOKO T cells produced IFN-γ after short-term restimulation *in vitro*. On a per-cell basis, P14.DOKO T cells tend to produce less IFN-γ compared to P14.WT T cells, as seen in a reduced MFI (Fig. 2 F, right panel). Differences in TNF-α production between all four P14 T cell populations were not observed (Fig. 2 G, right panel). Of note, more P14.IFNARKO, P14.IL-12RKO and P14.DOKO T cells produced IL-2 compared to P14.WT T cells after short-time restimulation *in vitro* (15% versus 5% of P14.WT T cells, Fig. S2 A), which correlates with the more prominent MPEC phenotype of these T cells. However, *ex vivo* killing activity of P14.WT, P14.IFNARKO and P14.IL-12RKO T cells was comparable as measured in a ^51^Cr-release assay. Interestingly, P14.DOKO T cells demonstrated a slightly reduced capacity to lyse peptide-loaded target cells (Fig. 2 E) with about 3 times more effector T cells needed for comparable cytolytic activity, although CD107a expression revealed no defect in degranulation capacity (Fig. S2 A).

Our results indicate that during VSV infection, CD8 T cells lacking type I IFN receptor are already reduced in their expansion. However, T cell expansion is even more limited if both signal 3 cytokines are lacking, indicating a synergistic effect of type I IFN and IL-12 as signal 3. Interestingly, at least one signal 3 cytokine is needed for the up-regulation of KLRG-1 and the development of full cytolytic effector functions, as CD8 T cells lacking both signal 3 cytokines are slightly impaired in IFN-γ production and cytolytic activity during VSV infection.

**Figure 3:**
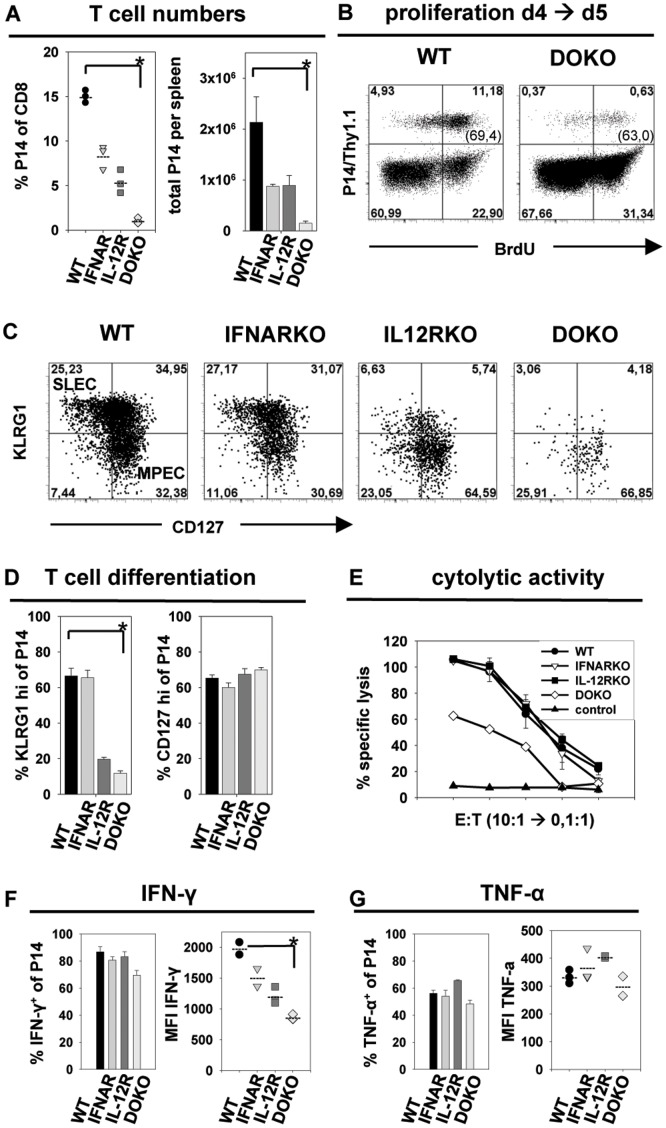
Analysis of P14 CD8 T cells in the context of a *rListeria_GP33_* infection. P14.WT, P14.IL-12RKO, P14.IFNARKO or P14.DOKO T cells were transferred into B6 mice followed by infection with *rListeria_GP33_*. Analysis of spleen cells at day 5.5 after infection is shown. ***A***, Relative and total P14 T cell numbers per spleen is shown. ***B***, Analysis of cell division by BrdU incorporation. After adoptive transfer, recipient mice were treated with 2 mg of BrdU i.p. on day 4. BrdU staining of P14.WT or P14.DOKO T cells gated on CD8^+^ T cells on day 5 in the spleen is shown. Numbers in brackets indicate percentages of BrdU^+^ cells of total P14 T cells. ***C***, Analysis of KLRG1 and CD127 expression on P14 T cells. Plots are gated on CD8^+^, Thy1.1^+^ (P14) cells. ***D***, Statistical analysis of T cell differentiation. Percentages of KLRG1 and CD127 positive P14 T cells of total P14 T cells are depicted. ***E***, Cytolytic activity of P14 T cells was determined five days post infection by a ^51^Cr-release assay. Gp33-41 peptide-loaded or adeno peptide-loaded EL-4 cells (control) were used as targets. Equal numbers of purified P14 T cells were used as effectors. Expression of IFN-γ ***(F)*** and TNF-α ***(G)*** of P14 T cells. Percentages of cytokine positive P14 T cells of total P14 T cells, as well as MFI of cytokine expression are indicated. Values are expressed as mean ± SEM (n = 3). *p<0,01. Results are representative of three independent experiments.

In contrast to the phenotype observed after VSV infection, P14 T cell populations that lack either IL-12 or type I IFN receptor already showed a reduced expansion after *Listeria* infection (Fig. 1 C). The lack of both signal 3 cytokine receptors further enhanced this phenotype, leading to drastically diminished expansion of P14.DOKO T cells. We analyzed transferred T cells in the spleen in more detail at day 5 after *Listeria* infection, the peak of expansion of P14.DOKO T cells (Fig. 1 C). P14.WT T cells showed the most prominent expansion in the spleen (14% of total CD8 T cells), followed by P14.IFNARKO and P14.IL-12RKO T cells (8% versus 5% of total CD8 T cells) and P14.DOKO T cells (1% of total CD8 T cells), with frequencies reduced by a factor of ∼14 compared to P14.WT T cells (Fig. 3 A). Of note, the difference in expansion of P14.DOKO to P14.WT T cells in the spleen is less pronounced compared to the 20 fold reduction observed in the blood. This disparity is probably due to the time point of analysis (day 5 in the spleen versus day 6 in the blood) and the fact that P14.WT cells strongly expand during day 5 and 6 after *Listeria* infection, whereas P14.DOKO T cells decline in numbers. BrdU incorporation experiments revealed that about 65% of P14.WT and P14.DOKO T cells divided between day 4 and day 5 (Fig. 3 B), indicating that the proliferative capacity of P14.DOKO T cells is intact and most probably impaired survival and not reduced proliferation might be the reason for lower P14.DOKO T cell numbers.

As previously published, CD8 T cells lacking IL-12 signaling fail to differentiate into SLEC during *Listeria* infection [11]. To investigate the role of type I IFN in the differentiation into effector cells, we analyzed KLRG1 and CD127 expression on P14.WT, P14.IL-12RKO, P14.IFNARKO and P14.DOKO T cells in the spleen day 5 after *Listeria* infection. P14.WT and P14.IFNARKO T cells up-regulated KLRG1 and down-regulated CD127 to the same extent, leading to the formation of SLEC and MPEC populations of comparable size (Fig. 3 C). About 60% of P14.WT and P14.IFNARKO T cells exhibited a strong expression of KLRG1, whereas only 5–15% of P14.IL-12RKO and P14.DOKO T cells were able to up-regulate KLRG1 (Fig. 3 D) indicating a profound defect in the differentiation toward an SLEC phenotype (Fig. 3 C and D). Of note all four P14 T cell populations up-regulated CD44 and down-regulated CD62L to the same extent (Fig. S2 B).

To investigate if the more prominent MPEC phenotype of P14.IL-12RKO and P14.DOKO T cells correlated with cytokine production, IFN-γ, TNF-α and IL-2 were stained intracellularly. About 80% of P14.WT, P14.IFNARKO and P14.IL-12RKO T cells produced IFN-γ after *Listeria* infection, but only about 70% of P14.DOKO T cells. Interestingly, P14.DOKO T cells exhibited strongly reduced levels of IFN-γ expression, as seen in the lower MFI, compared to P14.WT (Fig. 3 F right panel). In contrast, no differences in TNF-α and IL-2 expression were observed (Fig. 3 G right panel and Fig. S2 B). Furthermore, P14.DOKO T cells were strongly impaired in their *ex vivo* cytolytic effector functions, as 9 times more effector T cells were needed to obtain comparable cytolytic activity in a ^51^Cr-release assay (2 titration steps, Fig. 3 E) and a decreased degranulation capacity according to CD107a surface staining (Fig. S2 B), when compared to P14.WT, P14.IFNARKO and P14.IL-12RKO T cells. These results indicate that type I IFN compensates absent IL-12 signals in establishing cytolytic activity of CD8 T cells but does not replace a missing IL-12 signal for T cell expansion or the differentiation into SLEC. In addition, the reduced expansion and the preferential differentiation into MPEC correlated with lower IFN-γ production and reduced cytolytic activity of those T cells that lack both signal 3 cytokines in the context of a *Listeria* infection. Thus, presence or absence of signal 3 cytokines not only influences T cell expansion and survival but also differentially modulates cell fate and effector functions.

### Impaired SLEC Formation and Reduced Effector Functions Correlate with Altered Expression of Transcription Factors T-bet and Eomes

**Figure 4:**
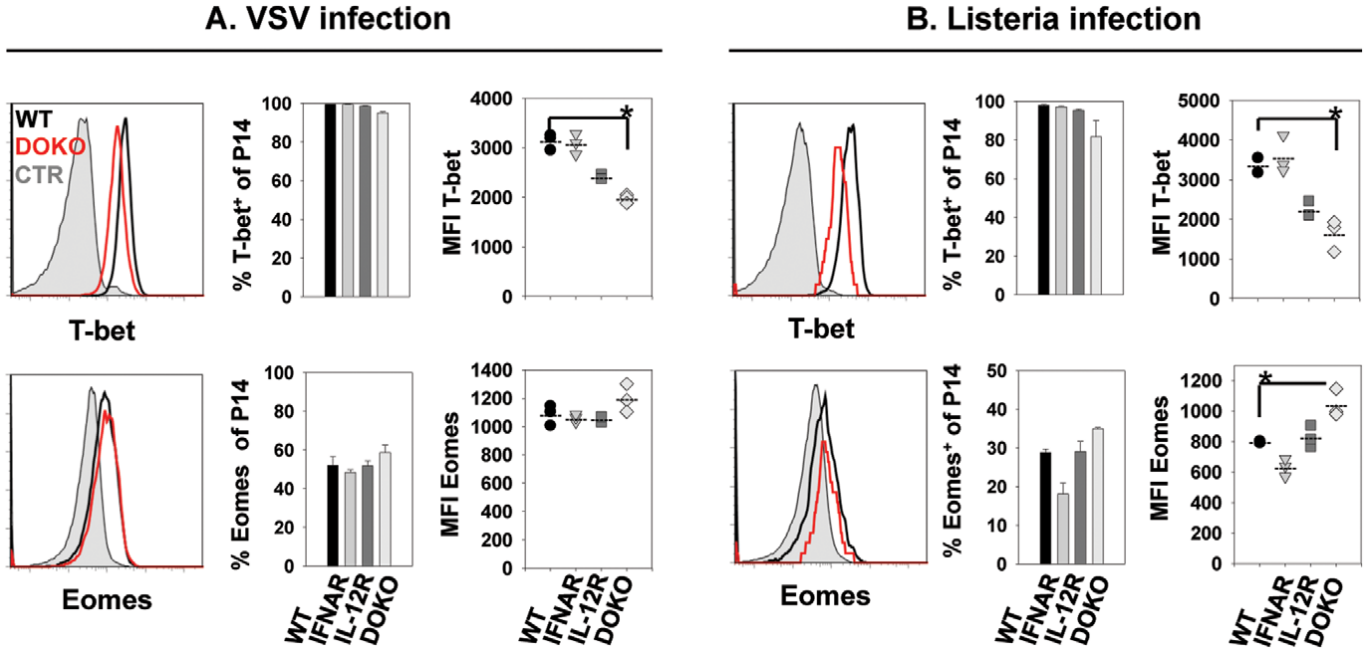
Expression of T-box transcription factors T-bet and Eomes after infections with rVSV_GP_ and *rListeria_GP33_* infection. P14.WT, P14.IL-12RKO, P14.IFNARKO or P14.DOKO T cells were transferred into B6 mice followed by infection with ***A,*** rVSV_GP_, or ***B,*** *rListeria_GP33_*. Expression of T-bet and Eomes was analyzed in the spleen on day 5 after infection. Histograms show intracellular expression of T-bet or Eomes in P14.WT (black line), P14.DOKO (red line) and naïve P14 CD8 T cells (filled curve) directly *ex vivo*. Percentages of T-bet or Eomes positive P14 T cells of total P14 T cells are indicated (middle panels). Mean fluorescence intensity (MFI) of T-bet or Eomes expression of P14 T cells is shown (right panels). p<0,01. Results are representative of two independent experiments.

We further analyzed the contribution of type I IFN and IL-12 signals to the transcriptional regulation of CD8 T cell differentiation thereby focusing on the two T-box transcription factors T-bet and Eomes. *In vivo,* after adoptive transfer and VSV infection, 90–100% of P14.WT, P14.IFNARKO, P14.IL-12RKO and P14.DOKO T cells expressed T-bet. However, on a per-cell basis, P14.IL-12RKO and P14.DOKO T cells exhibited reduced T-bet expression levels (as seen in the MFI, Fig. 4 A, upper right panel). Analysis of Eomes expression levels illustrated that, on a per-cell basis, P14.DOKO T cells expressed slightly more Eomes compared to P14.WT, P14.IFNARKO and P14.IL-12RKO T cells (MFI of Eomes on total P14, Fig. 4 A, lower right panel). After *Listeria* infection 90–100% of P14.WT, P14.IFNARKO, P14.IL-12RKO and P14.DOKO T cells expressed T-bet, although the expression level on a per-cell basis was reduced in P14.IL-12RKO T cells, and even more pronounced in P14.DOKO T cells (Fig. 4 B, upper right panel). Again, this lower expression level of T-bet in P14.DOKO T cells correlated with higher expression of Eomes in these cells (Fig. 4 B, lower right panel). These results support the notion that mainly IL-12 regulates T-bet expression. Type I IFN play a minor role since P14.IFNARKO T cells are only slightly impaired in T-bet expression during VSV and *Listeria* infection.

### The Pathogen-induced Inflammatory Milieu Determines Signal 3 Dependence of CD8 T Cells

**Figure 5:**
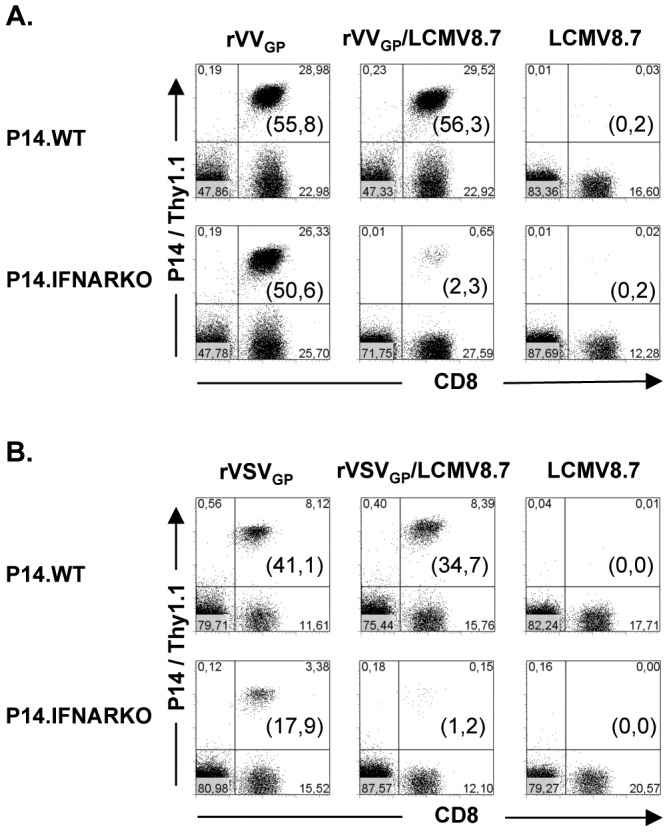
The pathogen-induced inflammatory milieu determines signal 3 dependence of CD8 T cells. P14.WT or P14.IFNARKO T cells were transferred into B6 mice followed by infection with ***A,*** rVV_GP_, rVV_GP_ and LCMV8.7 or LCMV8.7 alone or, ***B,*** rVSV_GP_, rVSV_GP_ and LCMV8.7 or LCMV8.7 alone. Analysis of peripheral blood lymphocytes at day 5 after infection is shown. Numbers in brackets indicate percentages of Thy1.1 positive of total CD8 T cells. Results are representative of two independent experiments.

The mechanistic basis for the different signal 3 dependence of T cells in the context of various infections is an open question. Diverse signal 3 requirements may be influenced by differences in the antigen load, the quality of antigen presentation (influenced by cell tropism of a pathogen) and the induced local inflammatory milieu. To address this question in more detail we established two experimental approaches, in which the delivery of the antigen-specific signal 1 and the co-stimulatory signal 2 are separated from the pathogen-induced inflammatory milieu. To this end we chose the LCMV infection model, since CD8 T cell expansion is strictly dependent on type I IFN as signal 3. First we established a co-infection model using rVV_GP_ expressing the GP33 epitope and LCMV8.7, a LCMV isolate that is not recognized by P14 T cells due to a mutation in the GP33 epitope. In this situation, transferred P14.WT and P14.IFANRKO T cells recognize their nominal GP33 epitope exclusively on rVV_GP_ but not on LCMV8.7 infected APCs. As shown in Figure 5 A (right panel) P14.WT and P14.IFANRKO T cells did not expand in the context of a LCMV8.7 infection, as they are not able to recognize the mutated GP33 epitope. After infection with rVV_GP_ both P14 T cell populations expanded vigorously (Fig. 5 A, left panel). However, in the context of a rVV_GP_/LCMV8.7 co-infection only P14.WT but not P14.IFANRKO T cells were able to expand (Fig. 5 A, middle panel). This indicates that CD8 T cells are strictly dependent on a type I IFN signal in a LCMV8.7-induced inflammatory milieu, even in a situation in which the antigen-specific signal 1 is delivered independently of the LCMV infection. Using a rVSV_GP_/LCMV8.7 co-infection we could confirm these findings. P14.WT and P14.IFANRKO T cells expanded after rVSV_GP_ but not after LCMV8.7 infection (Fig. 5 B, left and right panels). During rVSV_GP_/LCMV8.7 co-infection high frequencies of P14.WT T cells were detectable on day 5 post infection in the blood, whereas P14.IFNARKO T cell frequencies were drastically reduced at this time point (Fig. 5 B, middle panel). Thus, investigating two co-infection models which separated antigen-specific stimulation from the overall induced inflammatory milieu, we demonstrated that the dominant LCMV-induced inflammatory milieu determines the type I IFN dependence of the antigen-specific CD8 T cells, although LCMV was not involved in antigen-specific stimulation and the antigen-load and the quality of the antigen-presentation were not limited.

**Figure 6:**
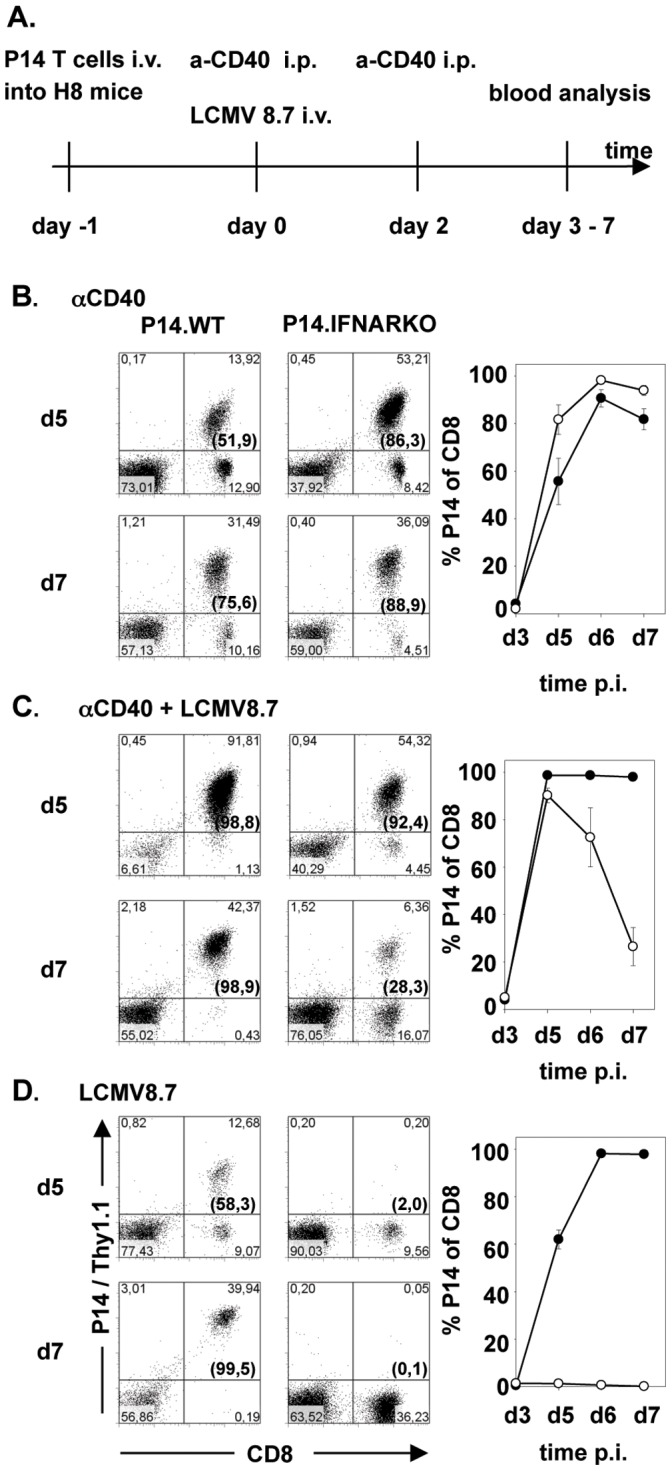
Infection independent T cell expansion can be suppressed by the LCMV-induced inflammatory milieu. A timeline of the experiment is shown in ***A***. P14.WT or P14.IFNARKO T cells were transferred into H8 mice at day −1, followed by ***B,*** treatment with anti-CD40 antibody alone (day 0 and day 2), ***C***, treatment with anti-CD40 antibody and infection with LCMV8.7 or ***D***, infection with LCMV8.7 alone. Expansion of transferred CD8 T cells at day 5 and day 7 after treatment and/or infection is shown in the blood. Numbers in brackets indicate percentages of Thy1.1 positive of total CD8 T cells. Kinetics in the right panels indicates the expansion of the transferred Thy1.1 T cells of total CD8 T cells from day 3 to day 7 in the blood. Values are expressed as mean ± SEM (n = 3). Results are representative of two independent experiments.

In a second experimental approach we separated antigen load and antigen presentation from the pathogen-induced inflammatory milieu in an even more radical way. We used H8 mice as recipient mice for P14 T cells (time line see Fig. 6 A). H8 mice ubiquitously express the GP33 epitope under the control of a MHC class I promoter and, thus, antigen load/presentation is defined and totally independent of a given infection. P14.WT and P14.IFANRKO T cells transferred into H8 mice recognized GP33 antigen but did not expand as they were rapidly tolerized [26]. After adoptive transfer and treatment of H8 mice with an agonistic anti-CD40 antibody, both P14.WT and P14.IFANRKO T cells expanded with comparable kinetics (Fig. 6 B). However, when anti-CD40 treatment was combined with a LCMV8.7 infection, P14.IFNARKO T cells initially expanded until day 5 and crashed dramatically thereafter, indicating that a direct type I IFN signal is a crucial survival factor for the already expanded T cells in this situation. In contrast P14.WT T cells expanded and T cell numbers stayed constant over the time of the experiment (Fig. 6 C). Again this demonstrates that T cells are strictly dependent on a signal 3 delivered by type I IFN in a LCMV-determined inflammatory milieu even in a situation where antigen-presentation is totally separated from the infection. The different behavior of the two P14 T cells populations is even more impressive after infection of H8 mice with LCMV8.7 alone. P14.WT T cells exhibited vigorous proliferation, as they recognize the endogenously expressed GP33 epitope on LCMV8.7 activated APCs. In striking contrast P14.IFNARKO T cells showed no expansion in the LCMV-induced inflammatory milieu, indicating the critical role of type I IFN as signal 3.

In summary, these experiments demonstrated that the signal 3 dependence of CD8 T cells is mainly determined by the pathogen-induced inflammatory milieu and not or to a less extent by the antigen load and/or the quality of the antigen presentation during LCMV infection.

## Discussion

In this study we tested the hypothesis that type I IFN and IL-12, two signal 3 cytokines, compensate each other in supporting CD8 T cell activation in the context of different infections. Using CD8 T cells that lack either type I IFN receptors, IL-12 receptors or both in four different infections, we could show a complex pattern of the requirement for those two third signals. Our results indicate that the infection typical inflammatory milieu dictates whether the expansion of CD8 T cells depends on type I IFN (LCMV), type I IFN and IL-12 (*Listeria*, VSV), or neither type I IFN nor IL-12 (VV). Interestingly, during VSV infection, type I IFN substitutes IL-12 as third signal, whereas during *Listeria* infection both cytokines are needed for expansion of CD8 T cells. Similar results were reported by Obar et al. in the OT-1 T cell adoptive transfer system, with the difference that IL-12 could not fully compensate a lack in type I IFN signaling for T cell expansion during a VSV infection [29]. Surprisingly, after VV infection, neither IL-12 nor type I IFN are critical for initial expansion of T cells, indicating that signal 3 cytokines are dispensable or a further signal 3 cytokine has to be postulated [30]. It was reported recently that IL-2, usually known as a costimulatory signal (signal 2), can support T cell activation similar to type I IFN and IL-12. High amounts of IL-2 were shown to enhance cytolytic activity of CD8 T cells and differentiation into effector CTL (CD127 low) [31], [32]. Additionally, IL-2 signaling induced Eomes, but not T-bet expression. The interplay of IL-2 and IL-12 was controversial: IL-12 prolonged IL-2 responsiveness by up-regulating CD25 but also repressed Eomes expression, thereby interfering with the ability of IL-2 to regulate certain genes. In addition, during LCMV infection, CD8 T cells expressing CD25 become highly activated effector CTL, whereas CD25 low cells formed the memory CTL pool [16]. It therefore remains to be investigated, whether IL-2 compensates for IL-12 and type I IFN in our model during VV infection.

Two recently published studies investigated T cell help dependence of CD8 T cell priming during VV infection. Results demonstrate that T helper cells promote CD8 T cell priming via two synergistic pathways, by inducing IL-12 production by DC after CD40/CD40L interaction and being a source of IL-2 by themselves [33]. In this setting, IL-12 alone was not sufficient to replace T cell help. Only the provision of IL-12 together with IL-2 substituted T cell help for CD8 T cell priming. In contrast to IL-12, high amounts of type I IFN were sufficient to compensate for the lack of T cell help, as CD8 T cells showed increased effector functions and expansion in the presence of type I IFN during VV infection [34]. This is in line with our data indicating that the signal 3 cytokines type I IFN and IL-12 are not fully redundant for activation of CD8 T cells and therefore only partially compensate each other in different infections. These data together indicate that CD8 T cell activation is dependent on a complex pattern of cytokines expressed during different infections. Furthermore, the amount of cytokine produced seems to play a critical role, since only high amounts of type I IFN were able to substitute for IL-12 signals.

Dependence of CD8 T cell activation on signal 3 cytokines in the context of VV infection is a matter of debate. We and others observed that the initial expansion of CD8 T cells is largely independent of IL-12 and type I IFN during VV infection and that T cell populations lacking one of the signal 3 receptors may decline slightly more pronounced when compared to P14.WT T cells [11], [35]. Reports by other groups indicate that CD8 T cell activation is dependent on a signal 3 delivered either by type I IFN or IL-12 [7], [33], [34]. The observed discrepancy might be due to different infection routes used in these studies. After i.v. injections of VV CD8 T cell activation was largely independent of signal 3 cytokines in our study, whereas after i.p. infections a signal 3 dependency was observed in other studies. However, performing i.v. and i.p. infection experiments with VV in parallel, we could not observe a considerable impact of the infection route on the signal 3 dependence for expansion of P14 T cells (Fig. S3). Therefore, some of the discrepancies may simply be due to the time point of analysis as CD8 T cells lacking IL-12, type I IFN receptors or both declined more pronounced after the peak of expansion compared to wild-type T cells. Since detailed kinetics of T cell expansions are lacking in other studies, the peak of expansion is not obvious and the time point of analysis might be located during contraction phase.

It was postulated that the formation of memory T cells is regulated directly by IL-12 and type I IFN [8], [36]. However, the findings are so far controversial, since on the one hand signal 3 cytokines were implicated to be needed for memory formation after VV or *Listeria* infection [8] while on the other hand IL-12 signals inhibited memory formation after *Listeria* infection [36]. The impact of signal 3 cytokines on the formation of memory T cells is difficult to interpret in cases where already a drastically reduced expansion of T cells in the acute phase was observed. In our model, nevertheless, memory T cells deficient in IL-12 signaling were detectable later than 30 days after *Listeria* infection [11]. Furthermore, in the context of VV infection memory T cells developed independently of a signal 3 (Fig. 1). This contrasts previously published results which demonstrated that OT-1 T cells lacking both type I IFN receptor and IL-12 receptor failed to form memory populations in response to VV and *Listeria* infections [8]. Additionally the expansion of OT-1 T cells during *Listeria* infections was largely independent on a direct type I IFN or IL-12 signal, whereas in our hands P14 T cells need signal 3 cytokines for expansion after *Listeria* infection. It is unclear which factor is the basis for this discrepancy. In the study mentioned above, OT-1 T cells deficient in the IL-12Rß1 chain were used, which lack both, IL-12 and IL-23 signals, whereas in the present paper we used P14 T cells deficient in the IL-12ß2 chain which exclusively lack IL-12 signals. Furthermore the use of different TCR transgenic T cells (varying in TCR avidity), infection with different recombinant VV and use of different application routes may have an impact on signal 3 dependence of T cell responses. However, in a recent study Obar et al demonstrated that OT-1 T cells also critically depend on both type I IFN and IL-12 for CD8 T cells expansion in the context of a *Listeria* infection [29], supporting our findings in the P14 T cell system.

To determine the contribution of type I IFN and IL-12 to the transcriptional regulation in CD8 T cell differentiation, we analyzed the expression of two T-box transcription factors T-bet and Eomes. Both transcription factors are induced in effector CD8 T cells and regulate IFN-γ expression and cytolytic activity [17], [37]. Because of their redundant roles, they can complement each other but also differentially regulate effector and memory CTL formation. T-bet was suggested to induce the transition of CD8 T cells into effector CTL, whereas Eomes was associated with memory formation during *Listeria* infection [19]. In addition, the expression of T-bet and Eomes is differentially regulated: While high T-bet expression levels are induced via TCR signaling and IL-12, expression of Eomes was associated with IL-2 signaling [32]. Since signal 3 cytokines influence the expression of CD25 on CD8 T cells they may indirectly influence expression of Eomes. In a further study type I IFN and IL-12 were shown to up-regulate or to maintain the expression of both transcription factors after stimulation *in vitro* [4]. Our *in vivo* model indicates that mainly direct IL-12 signals determine the high expression of T-bet whereas type I IFN have a modulating effect, as CD8 T cells lacking IL-12 signals but not type I IFN signals exhibited diminished expression levels compared to wild-type cells. The impact of type I IFN on regulation of T-bet is only evident if T-bet expression levels between P14.IL-12RKO and P14.DOKO T cells are compared, showing that T-bet expression in P14.DOKO T cells was further reduced. Of note, Eomes expression levels are exclusively enhanced if both cytokines, IL-12 and type I IFN, are missing, although Eomes expression preferentially increased relative to T-bet already if only IL-12 signals were missing. It is tempting to speculate that T-bet expression regulates Eomes, with high levels of T-bet repressing Eomes expression. In addition, the overall expression levels of Eomes were lower after *Listeria* infection compared to VSV infection, a finding that might be explained by higher IL-12 levels induced after *Listeria* infection which is in accordance with previous studies demonstrating that IL-12 mediates repression of Eomes [19]. Interestingly, the higher expression level of Eomes in P14.DOKO T cells is not sufficient to compensate the lack of T-bet in induction of effector functions, as suggested previously [17]. Thus, CD8 T cells lacking both signal 3 cytokines showed impaired cytokine production and cytolytic activity on a per-cell basis. In addition, KLRG1 expression was diminished in those cells. Our results therefore demonstrated for the first time a direct correlation of signal 3 availability and the expression levels of the T-box transcription factors T-bet and Eomes for CD8 T cell fate decisions and effector functions *in vivo*. During both, VSV and *Listeria* infection, T cells lacking type I IFN and IL-12 signals down-regulated T-bet and up-regulated Eomes, thereby showing a memory CTL phenotype, with high CD127 and low KLRG1 expression. In addition, these T cells were impaired in IFN-γ expression and exhibited reduced cytolytic activity. A recent study demonstrated that the early cytokine milieu induced by *Listeria* or VSV infection, differentially determines effector T cell differentiation into SLECs and MPECs. Additionally, these authors showed that strong inflammation and thus IL-12 production induced by CpG application promoted CD8 T cell differentiation into a SLEC phenotype during VSV infection. Interestingly, for the IL-12 independent SLEC differentiation of CD8 T cells during VSV infection the transcription factor Ebi3 was critically involved. It is tempting to speculate that IL-27 may be involved in the differentiation of CD8 T cell into a KLRG-1^high^/CD127^low^ phenotype and regarded as an alternative signal 3 cytokine [29]. Thus, our and published data strongly support the concept that a highly inflammatory milieu, mainly in form of high IL-12 cytokine levels, drives SLEC formation while inhibiting MPEC differentiation [12], [29], [38].

Besides the differential impact of signal 3 cytokines on the cell fate decision of effector CD8 T cells, we would like to emphasize the direct role of a signal 3 for survival of effector T cell in the context of a given infection. To analyze signal 3 requirements for T cell survival in an acute infection in more detail, we established two independent experimental settings, in which the delivery of the antigen-specific signal 1 and the co-stimulatory signal 2 are separated from the pathogen-induced inflammatory milieu. First, using co-infection experiments with rVV_GP_/LCMV8.7 and rVSV_GP_/LCMV8.7 we could demonstrate that the LCMV8.7-induced inflammatory milieu determines the signal 3 dependence of CD8 T cells. CD8 T cells deficient in type I IFN receptor were not able to expand in the context of a rVV_GP_ or rVSV_GP_ infection, when a LCMV typical inflammatory milieu is induced simultaneously. Of note, the LCMV8.7 isolate by itself is not recognized by P14 T cells, due to a mutation in the GP33 epitope and therefore will only determine the inflammatory milieu. Thus, in presence of a LCMV inflammatory milieu CD8 T cells are strictly dependent on a direct type I IFN signal for survival. This is supported by the fact that type I IFN receptor deficient CD8 T cells enter cell cycle and undergo cell divisions *in vivo* but are failing at clonal expansion [5]. Interestingly using such a co-infection approach, Wiesel et al could recently demonstrate that the LCMV-induced type I IFN is not only critical for CD8 T cell expansion but also for the differentiation into SLECs [39].

Transfer of P14.WT and P14.IFNARKO T cells into H8 mice further demonstrated the role of signal 3 as a survival factor for effector T cell. H8 mice ubiquitously express the GP33 epitope and therefore antigen load and presentation is independent of an infection. After activation of APC by application of an agonistic anti-CD40 antibody, both P14 T cell populations expanded to the same extent. However, if anti-CD40 treatment was combined with a LCMV8.7 infection P14.IFNARKO T cells initially expanded but then dramatically crashed. Thus, in the context of a LCMV-induced inflammatory milieu even already expanded CD8 T cells are strictly dependent on a direct type I IFN signal for survival. We thus think that signal 3 cytokines may have an important role as a kind of fail-safe mechanism to ensure effector T cell survival in a given inflammatory milieu.

In summary, our results help to clarify the requirements for signal 3 cytokines for CD8 T cell expansion, differentiation and survival in the context of different infections. We showed that type I IFN signals can partially compensate IL-12 signals in CD8 T cell expansion and cytolytic activity *in vivo*. However, IL-12 signals seem to regulate transcription factor expression, thereby influencing the cell fate decision of CD8 T cells. Most significant, the relevance of IL-12 and type I IFN as signal 3 for T cell differentiation and survival is determined by the pathogen-induced inflammatory milieu. This knowledge of the modulatory capacity of signal 3 cytokines on CD8 T cells may have important implications for the improvement of classical vaccination strategies as well as for the development of new approaches in therapeutic vaccination, in persistent infection and cancer.

## Supporting Information

Figure S1 **Adoptive Transfer with low numbers of P14 T cells.** 10^4^ Thy1.1^+^ P14.WT, P14.IL-12RKO, P14.IFNARKO or P14.DOKO T cells were transferred into B6 mice (Thy1.2^+^) followed by infection with rVSV_GP_, rVV_GP_, LCMV or r*Listeria*_GP33_. Kinetics of P14.WT (•) and P14.IFNARKO, P14.IL12RKO and P14. DOKO (○). T cells gated on CD8^+^ T cells in the blood at indicated time points. Values are expressed as mean ± SEM (n = 3). Results are representative of three independent experiments.
(TIF)

Figure S2 **Analysis of P14 T cell after VSV and Listeria infection.** P14.WT, P14.IL-12RKO, P14.IFNARKO or P14.DOKO T cells were transferred into B6 mice followed by infection with ***A,*** rVSVGP or ***B,*** *rListeriaGP33*. Analysis of CD44 and CD62L expression on spleen cells at day 5 after infection is shown. Plots are gated on CD8, Thy1.1 (P14) cells. Expression of IL-2 and CD107a of P14 T cells after short time restimulation *in vitro* is indicated. Percentages of cytokine positive P14 T cells of total P14 T cells, as well as MFI of cytokine expression are depicted. Values are expressed as mean ± SEM (n = 3). * p<0,01 (Student́s unpaired t-test). Results are representative of three independent experiments.
(TIF)

Figure S3 **Expansion of P14 T cells after i.v. or i.p. infection with rVVGP.** 10^5^ Thy1.1, P14.WT, P14.IL-12RKO, P14.IFNARKO or P14.DOKO T cells were transferred into B6 mice (Thy1.2) followed by either ***A,*** intravenously or ***B,*** intraperitoneally infection with rVVGP. Kinetics of P14.WT (•) and P14.IFNARKO, P14.IL12RKO and P14. DOKO (○). T cells gated on CD8+ T cells in the blood at indicated time points. Values are expressed as mean ± SEM (n = 3).
(TIF)
