# Supplementary figures and images for: Signal 3 Cytokines as Modulators of Primary Immune Responses during Infections: The Interplay of Type I IFN and IL-12 in CD8 T Cell Responses
Source: PLoS One. 2012 Jul 17;7(7):e40865. doi: 10.1371/journal.pone.0040865 (PMC3398954; doi:10.1371/journal.pone.0040865)

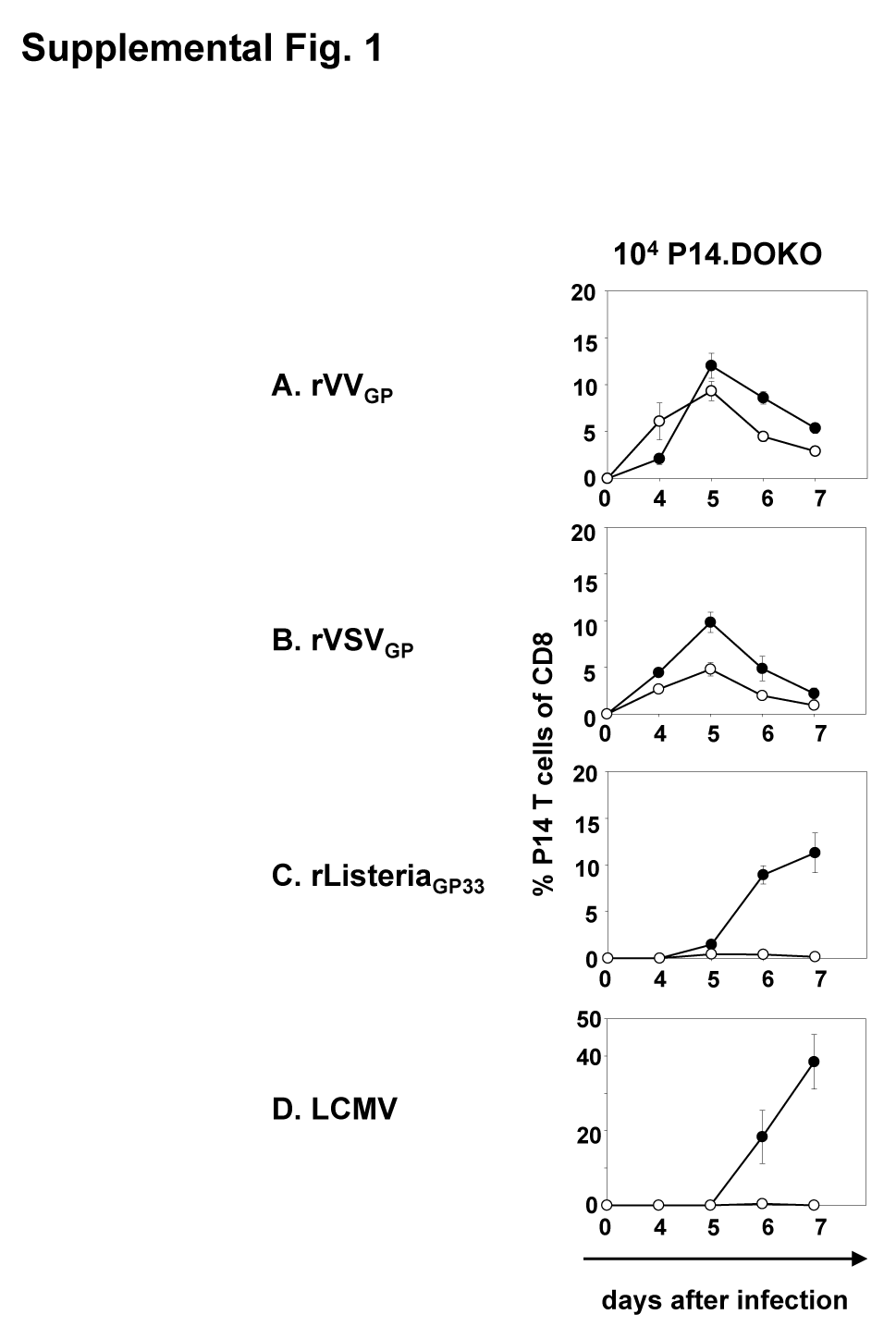

Supplement: Figure S1 — Adoptive Transfer with low numbers of P14 T cells. 104 Thy1.1+ P14.WT, P14.IL-12RKO, P14.IFNARKO or P14.DOKO T cells were transferred into B6 mice (Thy1.2+) followed by infection with rVSVGP, rVVGP, LCMV or rListeria GP33. Kinetics of P14.WT (•) and P14.IFNARKO, P14.IL12RKO and P14. DOKO (○). T cells gated on CD8+ T cells in the blood at indicated time points. Values are expressed as mean ± SEM (n = 3). Results are representative of three independent experiments. (TIF) [file pone.0040865.s001.tif]

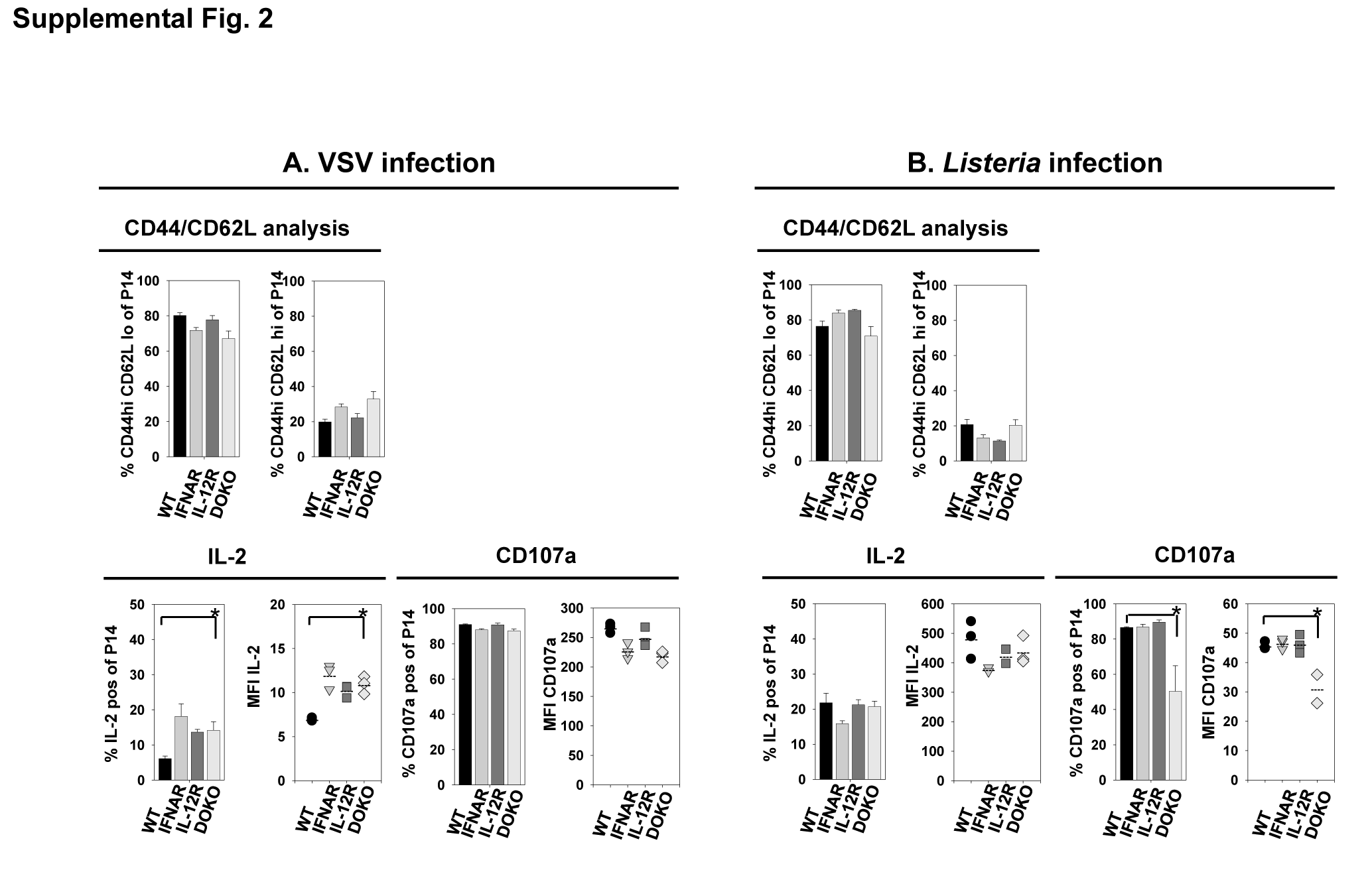

Supplement: Figure S2 — Analysis of P14 T cell after VSV and Listeria infection. P14.WT, P14.IL-12RKO, P14.IFNARKO or P14.DOKO T cells were transferred into B6 mice followed by infection with A, rVSVGP or B, rListeriaGP33. Analysis of CD44 and CD62L expression on spleen cells at day 5 after infection is shown. Plots are gated on CD8, Thy1.1 (P14) cells. Expression of IL-2 and CD107a of P14 T cells after short time restimulation in vitro is indicated. Percentages of cytokine positive P14 T cells of total P14 T cells, as well as MFI of cytokine expression are depicted. Values are expressed as mean ± SEM (n = 3). * p<0,01 (Student́s unpaired t-test). Results are representative of three independent experiments. (TIF) [file pone.0040865.s002.tif]

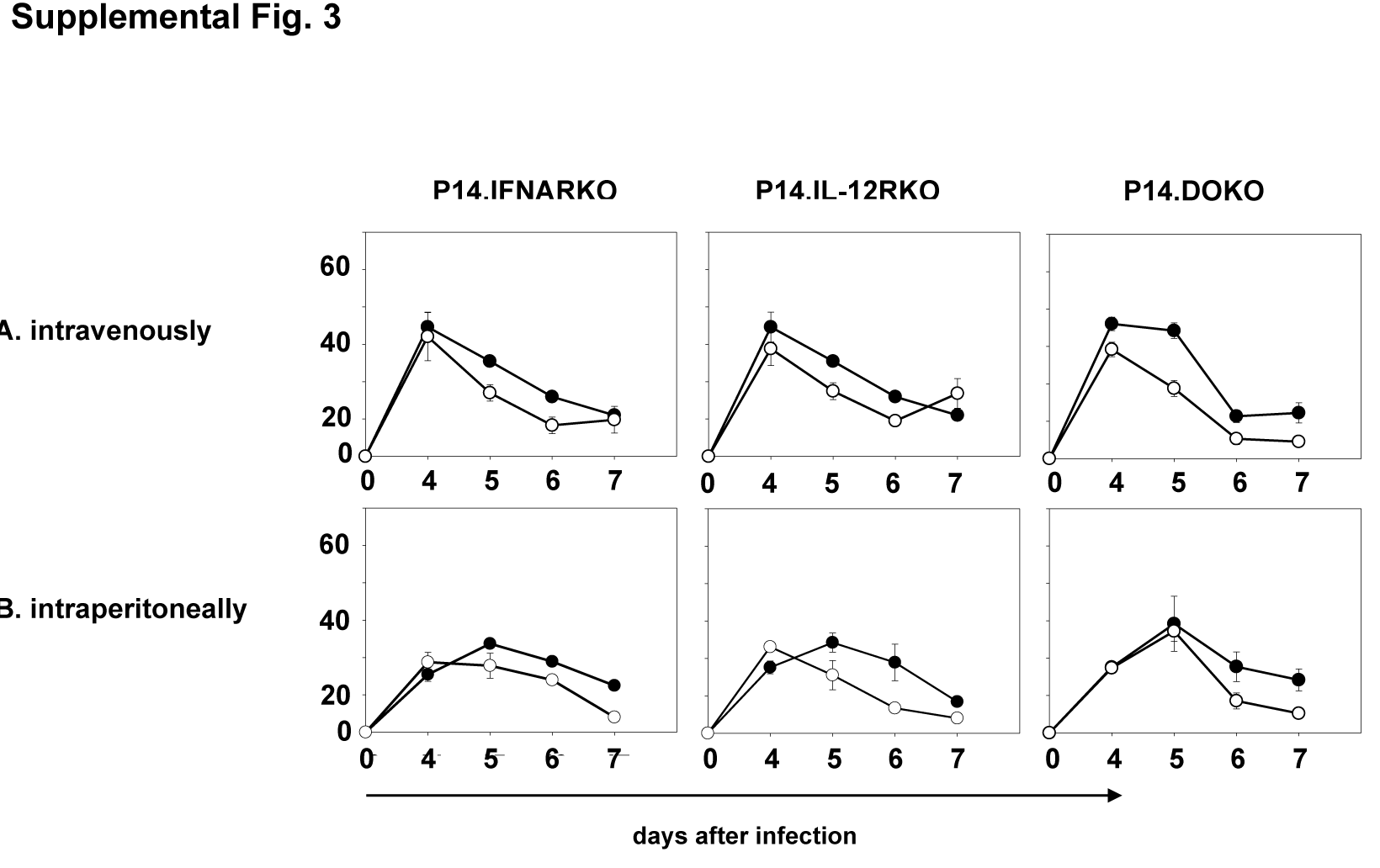

Supplement: Figure S3 — Expansion of P14 T cells after i.v. or i.p. infection with rVVGP. 105 Thy1.1, P14.WT, P14.IL-12RKO, P14.IFNARKO or P14.DOKO T cells were transferred into B6 mice (Thy1.2) followed by either A, intravenously or B, intraperitoneally infection with rVVGP. Kinetics of P14.WT (•) and P14.IFNARKO, P14.IL12RKO and P14. DOKO (○). T cells gated on CD8+ T cells in the blood at indicated time points. Values are expressed as mean ± SEM (n = 3). (TIF) [file pone.0040865.s003.tif]
